# Challenges of Treating an Acute Hepatitis C Virus Infection With Concurrent Seizure Treatment in a Free Clinic

**DOI:** 10.7759/cureus.25530

**Published:** 2022-05-31

**Authors:** Nicholas Blackmond, Joshua D Kanke, Tiffany Loh, Raymond Weitzman

**Affiliations:** 1 Internal Medicine, Gary Burnstein Community Health Clinic, Pontiac, USA; 2 Pharmacy, College Pharmacy, Colorado Springs, USA; 3 Internal Medicine, Oakland University William Beaumont School of Medicine, Rochester Hills, USA; 4 Rheumatology, Gary Burnstein Community Health Clinic, Pontiac, USA

**Keywords:** uninsured patients, anti-epileptic drug, direct-acting antiviral, hepatitis c management, hepatitis c (hcv) infection

## Abstract

Currently, there is limited data evaluating the coadministration of first-generation anticonvulsants for epilepsy treatment and direct-acting antivirals (DAAs) for the treatment of hepatitis C virus (HCV) infection. There is a potential risk of suboptimal DAA serum concentrations that could potentially lead to HCV treatment failure. In this report, we describe the case of an uninsured, non-Hispanic Black male in his mid-40s with a history of generalized epilepsy that was managed with phenytoin 100mg twice a day and divalproex 500mg extended-release once daily. He was diagnosed with an acute hepatitis C viral infection with a genotype of 1a. Due to the viral genotype, treatment naivety, and lack of cirrhosis*, *the recommended treatment is to start glecaprevir/pibrentasvir, but the concomitant use of phenytoin and glecaprevir/pibrentasvir is not recommended due to a drug-drug interaction that could lead to subtherapeutic DAA levels and possible treatment failure. Through shared decision-making and close follow-up, we slowly weaned the patient off phenytoin, replaced it with levetiracetam, and started glecaprevir/pibrentasvir. We successfully eradicated the patient’s HCV infection, and no breakthrough seizures were reported. Although an unprecedented case and with the limited data evaluating the coadministration of DAAs and treatment of acute HCV infection, we were able to successfully treat and achieve full remission of the HCV infection. By virtue of this case report, we hope to encourage others to report similar cases and spread awareness regarding the difficulties in management.

## Introduction

Hepatitis C virus (HCV) is a positive-sense, single-stranded, enveloped ribonucleic acid (RNA) virus with seven major genotypes. Acute HCV infection is diagnosed by detectable HCV RNA with undetectable HCV antibodies or detectable HCV RNA and detectable HCV lab with documentation of negative tests within the prior six months [1]. A total of 55%-85% of patients do not clear the virus and go on to develop chronic hepatitis C, with possible clinical manifestations of hepatic fibrosis, cirrhosis, hepatocellular carcinoma, sicca syndrome, cryoglobulinemia, glomerulonephritis, and porphyria cutanea tarda [1].

Direct-acting antivirals (DAAs) for hepatitis C were first approved by the Food and Drug Administration (FDA) in 2011 with peginterferon alfa and ribavirin. Since 2011, numerous DAAs have been developed and are highly effective, and peginterferon alpha and ribavirin have become less widely used [1]. DAAs affect viral replication and consist of NS3/4A protease inhibitors, NS5A replication complex inhibitors, and NS5B polymerase inhibitors. Glecaprevir and pibrentasvir are taken in combination. Glecaprevir is a NS3/4A protease inhibitor that prevents viral polyproteins from maturing, and pibrentasvir is a NS5A replication complex inhibitor that prevents viral RNA replication [1]. Glecaprevir is a substrate of CYP450, CYP3A4, and drug transporter P-glycoprotein (P-GP), and pibrentasvir is a substrate of P-GP [1,2]. First-generation anticonvulsants (phenytoin, carbamazepine, phenobarbital) are potent inducers of CYP450 enzymes and drug transporters [3]. Thus, administering glecaprevir/pibrentasvir with first-generation anticonvulsants can significantly decrease plasma concentrations of glecaprevir/pibrentasvir [1].

## Case presentation

A 41-year-old non-Hispanic Black male with a primary medical history of generalized epilepsy presented to a free clinic for routine follow-up. The patient had been successfully treated by a volunteer neurologist for his generalized epilepsy with phenytoin 100mg twice a day and divalproex extended-release 500mg once daily. After establishing care in 2018, he had been seizure-free. The patient reported compliance with medications without any adverse reactions. At the time of the visit, the patient reported drinking approximately a six-pack beer every other week, constipation, and diffuse abdominal pain. Routine serology, including a metabolic panel, was drawn. The metabolic panel, as shown in Table 1, revealed a slight increase in aspartate aminotransferase (AST) levels. The patient’s past medical history was negative for drug use, any breakthrough seizures or the use of any other medications, or hepatitis infection; he had no personal or family history of thyroid disease. He spent a brief period in jail for 60 days in late 2019 and completed blood work after release, and was told everything was normal. Unfortunately, we were unable to obtain any records from the county jail.

**Table 1:** Complete metabolic panel of the patient BUN: blood urea nitrogen; GFR: glomerular filtration rate; AST/SGOT: aspartate aminotransferase/serum glutamic-oxaloacetic transaminase; ALT/SGPT: alanine aminotransferase/serum glutamate-pyruvate transaminase *Out of range

Blood test	Value	Reference range
Sodium	139 mEq/L	135–144
Potassium	3.9 mEq/L	3.5–5.3
Glucose	76 mg/dL	70–99
BUN	11 mg/dL	7–25
Creatinine	0.75 mg/dL	0.60–1.20
GFR - Black	139 mL/min	>60
GFR - other	115 mL/min	>60
Alkaline phosphatase	62 U/L	27–120
Total bilirubin	1.0 mg/dL	0.3–1.0
AST/SGOT	51 U/L*	13–39
ALT/SGPT	38 U/L	7–52
Total protein	7.2 g/dL	6.1–7.9

We repeated a hepatic panel and added pancreatic enzymes to rule out acute pancreatitis, given the patient's diffuse abdominal pain and moderate alcohol use (Table 2). The hepatic panel did not show any abnormalities, and the AST level returned to normal. We found an elevated amylase level at 155 U/L, but a normal lipase level. The patient's diffuse abdominal pain had resolved after a bowel movement, and in the absence of fevers, nausea, vomiting, or decreased oral intake, we believe the amylase levels were falsely elevated. We repeated tests for pancreatic enzymes a week later, and both amylase and lipase levels were within normal limits. Muscle disorder was considered given the patient's history of seizures but was less likely as he was free of seizures for more than 12 months. Thyroid disease was considered, but there was no family history of thyroid disorders and the patient had no indication of thyroid disease.

**Table 2:** Hepatic panel and pancreatic enzyme levels of the patient AST/SGOT: aspartate aminotransferase/serum glutamic-oxaloacetic transaminase; ALT/SGPT: alanine aminotransferase/serum glutamate-pyruvate transaminase *Out of range

Blood test	Value	Reference range
Albumin	4.2 g/dL	3.5–4.8
Alkaline phosphatase	70 U/L	38–126
Bilirubin, total	0.9 mg/dL	0.0–1.6
AST/SGOT	34 U/L	15–41
ALT/SGPT	25 U/L	10–63
Protein	7.5 g/dL	6.1–7.9
Amylase	155 U/L*	28–100
Lipase	30 U/L	22–51

We ordered an acute hepatitis panel to evaluate for acute viral/alcoholic hepatitis, given the patient’s moderate alcohol use and time spent in jail. We ordered a liver ultrasound, but unfortunately it was not obtained as the patient was uninsured and unable to afford the cost to have the ultrasound performed. The acute hepatitis panel showed a positive hepatitis C antibody level (Table 3). A subsequent HCV quantitative molecular study was performed.

**Table 3:** Acute hepatitis panel IgM: immunoglobulin M

Blood test	Result	Reference
Hepatitis A antibody, IgM	Negative	Negative
Hepatitis B surface antigen	Negative	Negative
Hepatitis B core antibody, IgM	Negative	Negative
Hepatitis C antibody	Positive	Negative

The hepatitis C antibody third-generation enzyme immunoassay (EIA) test came back positive. The subsequent qualitative HCV result was positive, which, by using polymerase chain reaction (PCR), has a limit of detection of 12 IU/mL. HCV quantitative was 6,800,572 IU/mL, HCV quantitative log was 6.83 log IU, and the hepatitis C virus genotype was 1a (Table 4). Genotyping was determined by reverse transcription, PCR amplification, and electrochemical detection of the five untranslated and core regions of the HCV genome.

**Table 4:** HCV quantitative molecular study HCV: hepatitis C virus; Quant: quantitative *Out of range

Blood test	Value/result	Reference
HCV qualitative	Detected	Not detected
HCV Quant result	6,800,572 IU/mL*	<12
HCV Quant log result	6.83 log IU	<1.08
Hepatitis C virus genotype	1a	

Given the nature of a free clinic and that all our patients are low-income, without insurance, refugees, or homeless, acquiring specific prescriptions is difficult and typically involves patients applying to pharmaceutical companies that can take weeks to months before being approved. We took into account that while it is generally suggested that patients are treated with antiviral therapy in acute HCV infection rather than waiting six months to determine whether a chronic infection has been established, and given the time it would take to receive the medication, we opted to start treatment immediately. There are four medications that are recommended in the treatment of HCV genotype 1a without cirrhosis: ledipasvir/sofosbuvir 90-400mg, sofosbuvir/velpatasvir 400-100mg, elbasvir/grazoprevir 50-100mg and glecaprevir/pibrentasvir 100-40mg. Fortunately, we advocated for our patient and received glecaprevir/pibrentasvir 100-40mg. There was a delay in receiving this medication due to the requirements and processing time of the manufacturer and their patient assistance program. By the time we received glecaprevir/pibrentasvir, it was discovered that since the patient was on phenytoin, the concomitant use of glecaprevir/pibrentasvir was not recommended. These interactions have not been fully understood, but phenytoin’s induction of CYP3A4 and P-glycoprotein, an enzyme and transporter involved in glecaprevir/pibrentasvir metabolism and distribution, could lead to decreased levels of glecaprevir/pibrentasvir.

Ultimately, through shared decision-making, considering the difficulties of obtaining specific antiviral medications and the urgency to begin treatment, we adjusted his anticonvulsant regimen, shown in Table 5. The patient was instructed to take levetiracetam 500mg twice a day for one week and increase it to 1000mg twice a day for the second week. After five days of beginning levetiracetam, the patient was instructed to reduce his phenytoin dose from 200 to 100mg for one week. After seven days, the patient was told to stop the phenytoin and begin glecaprevir/pibrentasvir 100-40mg at 300-120mg daily five days after the last dose of phenytoin. Glecaprevir/pibrentasvir at 300-120mg daily was continued for eight weeks.

**Table 5:** Prescription regimen adjustment schedule tab(s): tablet(s)

	Prescription			
Week	Levetiracetam 500mg	Phenytoin 100mg	Depakote 500mg	Glecaprevir/pibrentasvir 100-40mg
Week 1; Day 1	One tab twice a day	Two tabs daily	One tab daily	
Week 1; Day 2	One tab twice a day	Two tabs daily	One tab daily	
Week 1; Day 3	One tab twice a day	Two tabs daily	One tab daily	
Week 1; Day 4	One tab twice a day	Two tabs daily	One tab daily	
Week 1; Day 5	One tab twice a day	Two tabs daily	One tab daily	
Week 1; Day 6	One tab twice a day	One tab daily	One tab daily	
Week 1; Day 7	One tab twice a day	One tab daily	One tab daily	
Week 2; Day 1	Two tabs twice a day	One tab daily	One tab daily	
Week 2; Day 2	Two tabs twice a day	One tab daily	One tab daily	
Week 2; Day 3	Two tabs twice a day	One tab daily	One tab daily	
Week 2; Day 4	Two tabs twice a day	One tab daily	One tab daily	
Week 2; Day 5	Two tabs twice a day	One tab daily	One tab daily	
Week 2; Day 6	Two tabs twice a day	Stop - discontinue	One tab daily	
Week 2; Day 7	Two tabs twice a day	Discontinue	One tab daily	
Week 3; Day 1	Two tabs twice a day	Discontinue	One tab daily	
Week 3; Day 2	Two tabs twice a day	Discontinue	One tab daily	
Week 3; Day 3	Two tabs twice a day	Discontinue	One tab daily	
Week 3; Day 4	Two tabs twice a day	Discontinue	One tab daily	Start (8 weeks) - three tabs daily
Week 3; Day 5	Two tabs twice a day	Discontinue	One tab daily	Three tabs daily
Week 3; Day 6	Two tabs twice a day	Discontinue	One tab daily	Three tabs daily
Week 3; Day 7	Two tabs twice a day	Discontinue	One tab daily	Three tabs daily

We monitored the patient closely for any breakthrough seizures, complications, and compliance over the next six months. The patient reported compliance and no adverse reactions. We re-evaluated for virologic response to treatment 12 weeks following cessation of therapy that demonstrated a complete resolution of detectable HCV RNA. The patient continued levetiracetam extended-release 2000mg daily and divalproex extended-release 500mg daily for seizure management.

## Discussion

The seroprevalence of HCV in the imprisoned population varies among states; however, it is still elevated compared to that in the general population. In 2006, about 17.4% of prisoners were estimated to be HCV antibody positive. Disproportionally, the incarcerated population constituted 28.5%-32.8% of all HCV cases in 2006 [4]. Approximately 16.1% (95% confidence interval [CI], 14.5-17.9) prisoners were estimated to be seropositive for HCV antibodies from 2013 to 2016. The HCV antibody prevalence in the general population was estimated to be 1.5% (95% CI, 1.3-1.8) [5]. In addition to the increased prevalence, the risk of mortality is also affected. In a study of incarcerated hospitalized patients in Massachusetts from 2011 to 2016, HCV was associated with increased mortality (hazard ratio, 1.61; 95% CI, 1.17-2.21) [6]. Other risk factors for HCV-related mortality include ethnicity, poverty, and level of educational achievement [7].

Recently incarcerated patients also have a higher likelihood of being uninsured [8]. Patients are more likely to avoid seeking care if they are uninsured and if they would experience great financial burdens, which is another factor [9]. In relation to HCV, DAAs are extremely expensive. In September 2015, the median price of a course of ledipasvir/sofosbuvir (Harvoni) was $63,509 [6]. This is where free clinics can bridge the gap. Social determinants of health are described by the World Health Organization as “non-medical factors that influence health outcomes.” One of the social determinants of health that free clinics can satisfy is quality healthcare services. Yet, free clinics are perceived as providing poor quality care due to various factors such as poor continuity of care and little or no specialist access [10]. This case showcases how free clinics can have excellent continuity of care and specialist access (internal medicine, neurology, and gastroenterology). Free clinics can provide access to healthcare and medications to patients who would otherwise delay or avoid seeking healthcare.

Yet, access to DAAs is difficult. As mentioned above, the median cost of ledipasvir/sofosbuvir in 2015 was quite prohibitive. From 2007 to 2009, a randomized trial evaluated the use of a transitions clinic versus expedited primary care for recently incarcerated patients. In the baseline characteristics, the study included the amount of liquid assets that were available to those patients [4-6]. Adjusted for inflation, those patients could only finance, at most, half of a course of ledipasvir/sofosbuvir. Even though the free clinic was able to help the patient obtain access to glecaprevir/pibrentasvir through AbbVie’s patient assistance program, attempting to follow current guidelines is occasionally difficult due to the restrictive costs of pharmacotherapy.

The pathophysiology of an active HCV infection lends itself to pharmacotherapeutic eradication from DAAs. The typical route of HCV transmission is through infected blood, such as IV drug use, high-risk sexual activity, or occupational exposure. There are higher rates of infection in men and incarcerated patients. Non-Hispanic Blacks historically have higher rates of chronic infection compared to Caucasians and Hispanic Whites [8]. Human hepatocytes are the primary target for HCV replication. HCV entry into a target cell is due to the action of HCV pseudoparticles and is pH- and clathrin-dependent. HCV RNA genome is post-translationally cleaved into 10 polypeptides, divided into structural and non-structural (NS) proteins. NS proteins, the proteases and RNA polymerase, are the site of action of DAAs, which lead to the prevention of HCV replication and eventual cure [2].

Due to DAAs being a substrate of CYP3A4 and P-GP, induction of these enzymes and transports could lead to detrimental effects on HCV therapy. Many first-generation antiepileptics are potent inducers of these systems. Even though this poses challenging decision-making, there is little guidance on management. There have been reports on preemptively increasing the dose of DAAs, continuing treatment without medication modification but careful monitoring, and adjustment of seizure therapy to avoid drug-drug interactions, such as ours [5].

## Conclusions

The management of administering direct-acting antivirals for hepatitis C virus and first-generation antiepileptics is still relatively unknown due to relatively few cases reported and the novel nature of DAAs in HCV treatment. Our case provides guidance on how coadministering these medications can be managed, but more research must be conducted to determine the optimal management course for patients on antiepileptics requiring HCV treatment. Additionally, our case highlights how a free clinic navigated these treatment challenges while advocating for a patient’s access to effective but typically financially burdensome treatment.
